# Topical Ocular Anti-TNFα Agent Licaminlimab in the Treatment of Acute Anterior Uveitis: A Randomized Phase II Pilot Study

**DOI:** 10.1167/tvst.11.6.14

**Published:** 2022-06-15

**Authors:** Theodore A. Pasquali, Melissa M. Toyos, David B. Abrams, David K. Scales, John W. Seaman, Georges Weissgerber

**Affiliations:** 1 SoCal Eye, Lakewood Eye Physicians, Lakewood, CA, USA; 2 Toyos Clinic, Nashville, TN, USA; 3 San Antonio Eye Center, San Antonio, TX, USA; 4 Retina and Uveitis Consultants of Texas, San Antonio, TX, USA; 5 Novartis Pharmaceuticals Corporation, Fort Worth, TX, USA; 6 Novartis Institutes for Biological Research, Basel, Switzerland

**Keywords:** anterior uveitis, topical treatment, anti-TNFα, licaminlimab, short-chain antibody fragment

## Abstract

**Purpose:**

Licaminlimab is a new anti-TNFα antibody fragment for topical ocular application. This phase II study assessed the tolerability, treatment effect, and pharmacokinetics of licaminlimab in acute anterior uveitis (AAU).

**Methods:**

In this multicenter, randomized, parallel-group, double-masked study, 43 adult patients with non-infectious AAU and Standardization of Uveitis Nomenclature (SUN) anterior chamber (AC) cell score of 2+ or 3+ were randomized (3:1 ratio) to licaminlimab (60 mg/mL, 8 drops/day for 15 days, 4 drops/day for 7 days, then matching vehicle for 7 days) or dexamethasone eye drops (8 drops/day for 15 days, tapering to 1 drop/day over 14 days). The primary efficacy end point was clinical response (≥2-step decrease in AC cell grade at day 15). A treatment effect was considered as established if the lower limit of the 95% posterior interval of the responder rate was >30%. Serum levels of licaminlimab were determined.

**Results:**

The day 15 response rate for licaminlimab was 56%; the lower bound of the 95% credible interval was 40% (i.e. >30%), demonstrating a treatment effect according to prespecified criteria. By day 4, 36% of licaminlimab-treated patients were responders; 76% had an AC cell grade of 0 on ≥1 post-treatment visit. The day 15 dexamethasone response rate was 90% (no inferential between-arm comparison was planned). Both treatments were well-tolerated. Intraocular pressure increased from baseline with dexamethasone but not licaminlimab. Licaminlimab was undetectable in serum in most patients.

**Conclusions:**

Licaminlimab is the first biologic demonstrated to have a treatment effect on an intraocular condition with topical ocular application. The trial met its primary objective and the observed responder rate for licaminlimab was 56.0%. Ocular administration of licaminlimab was well-tolerated in adult subjects with AAU for up to 35 days.

## Introduction

Non-infectious forms of uveitis include a highly heterogeneous group of conditions, with or without associated systemic disorders, that share an underlying immunological etiology.^1^ Anterior uveitis is the most prevalent type of uveitis and may be associated with visual impairment or, more rarely, blindness^2^; acute anterior uveitis (AAU) is the most common form of the disease,^3^ representing up to 49% of uveitis cases.^4^ Corticosteroids are the current mainstay of therapy, usually administered topically, although peri-ocular or systemic treatment may be used, depending on the extent and severity of disease.^1^^,^^5^ Corticosteroid therapy, whether topical, periocular, or systemic, can lead to increased intra-ocular pressure (IOP), which increases the risk of glaucoma,^6^^,^^7^ and cataracts.^8^ The use of systemic corticosteroids to treat ocular inflammation, especially in long-term therapy, is also associated with non-ocular safety issues, including diabetes mellitus, hypertension, peptic ulcer disease, osteoporosis, myopathy, and pancreatitis.^9^

The inflammation observed in anterior uveitis is mediated by T-cells and pro-inflammatory cytokines,^10^ and this pathology is targeted by a range of systemic treatments.^9^^,^^10^ The pro-inflammatory cytokine tumor necrosis factor α (TNFα) has been found at elevated levels in the aqueous humor of patients with uveitis,^11^ and TNFα blockade shows efficacy in animal models of uveitis.^12^ TNFα antagonists, for example, infliximab, adalimumab, certolizumab, and golimumab, are widely used in the treatment of a number of autoimmune and inflammatory conditions, including juvenile idiopathic arthritis, psoriasis and psoriatic arthritis, ulcerative colitis, rheumatoid arthritis, axial spondyloarthritis, ankylosing spondylitis, Crohn's disease, and ulcerative colitis. Some of these conditions are associated with uveitis.

Adalimumab is approved for the treatment of uveitis in Europe (chronic non-infectious anterior uveitis in patients from 2 years of age who have had an inadequate response to or are intolerant to conventional therapy, or in whom conventional therapy is inappropriate)^13^ and in the United States (non-infectious intermediate, posterior, and panuveitis in adults and children 2 years of age and older),^14^ and infliximab is approved in Japan for treatment of refractory uveoretinitis associated with Behçet's disease.^15^ Off-label use of other systemic TNFα antagonists has also been reported,^16^ and expert panel recommendations published in 2014^17^ suggest that infliximab and adalimumab could be used to treat a number of inflammatory ocular conditions, including several forms of uveitis. Whereas effective in the treatment of some forms of uveitis, current systemic TNFα antagonists are associated with significant safety issues, notably increased risks of serious infections, including tuberculosis, some types of malignancy, and demyelinating disorders.^13^^,^^18^^–^^20^

Topical ocular treatment with TNFα antagonists could potentially avoid systemic exposure and its associated risks. However, most currently available TNFα antagonists (infliximab, adalimumab, and golimumab) are whole monoclonal antibodies. Due to their biophysical properties, in particular their high molecular weights of approximately 150 kDa (whole IgG molecules), most of the marketed TNF-alpha antagonists would not be expected to penetrate ocular tissues following topical application.^21^^,^^22^

Single chain antibody fragments (scFvs), include only the variable domains from both light and heavy chains, and have different pharmacokinetic properties to full IgG molecules.^23^ In vitro experiments demonstrated that scFvs penetrate corneal tissue and appear to do so more effectively than Fab fragments.^24^ Topically applied anti-human TNFα scFv fragments have been shown to penetrate the anterior and posterior segments of the eye and reach therapeutic levels in rabbit models, with low systemic exposure.^25^^,^^26^ A study in patients undergoing cataract surgery or vitrectomy found that topical ocular application of the anti-human TNFα scFv fragment ESBA105 prior to surgery resulted in therapeutic concentrations in the aqueous humor, with a mean molar excess over intraocular TNFα ranging from 96-fold to 359-fold. Little or no penetration of the posterior segment (based on vitreous humor levels) was observed, and systemic exposure was very low.^27^

Licaminlimab (Oculis SA, Lausanne, Switzerland), formerly known as OCS-02, LME636, and ESBA1622, is an scFv that binds human TNFα. Here, we describe a phase II pilot study with topical ocular licaminlimab in the treatment of acute anterior uveitis.

## Methods

### Study Design

A randomized, active-controlled, parallel-group, multicenter design was used. The study was conducted at 10 sites in the United States. Patient eligibility criteria included age ≥18 years; diagnosis of non-infectious AAU in at least one eye; and Standardization of Uveitis Nomenclature (SUN) anterior chamber (AC) cell score of 2+ or 3+ in at least 1 eye. Key exclusion criteria were: AC cell score of 4+ or hypopyon; intermediate, posterior, or panuveitis in either eye; active or suspected active viral, bacterial, or fungal keratoconjunctival disease in either eye, or recent history (within 2 months of use of the study drug) thereof; active corneal abrasion or ulceration in either eye, or history thereof; and administration of systemic biologic anti-cytokines including TNFα inhibitors or immunosuppressive therapy within 2 months of use of the study drug. Exclusion criteria specifically relating to corticosteroid use were: administration of stable systemic corticosteroid doses equivalent to >10 mg daily prednisone within 2 months of use of the study drug; new use or change in dosage of any corticosteroid (including inhaled, nasal, or dermatological) within 2 weeks of use of the study drug; periocular injection (either or both eyes) of any corticosteroid solution within 2 weeks prior to use of the study drug, or of any depot corticosteroids within 6 weeks prior to use of the study drug; intravitreal injection of corticosteroid implants within 2.5 years of use of the study drug; use of more than the equivalent of 1 day of treatment (e.g. 8 drops of prednisolone 1%) of any topical corticosteroid or nonsteroidal anti-inflammatory drugs in the study eye (or in both eyes if both were to be treated) within 2 weeks of use of the study drug.

On day 1, patients were screened and randomized in a 3:1 ratio to licaminlimab or dexamethasone eye drops, and treatment was started. A randomization list was produced by the Sponsor using a validated system that automated the random assignment of treatment arms to randomization numbers in the specified ratio. Dexamethasone was the corticosteroid chosen as an active comparator in order to facilitate masking, as it was available as an ophthalmic solution rather than a suspension. Patients randomized to licaminlimab received 60 mg/mL ophthalmic solution, 8 drops/day for 15 days, then 4 drops/day for 7 days, followed by matching vehicle (2 drops/day for 4 days, then 1 drop/day for 3 days) to match the initial tapering of the dexamethasone arm. Dose selection was based on a preceding pilot phase II clinical study on the treatment of anterior acute uveitis with the surrogate molecule ESBA105 and the predicted maximum suppression of TNFα that could be achieved. Patients randomized to dexamethasone received 0.1% ophthalmic solution, 8 drops/day for 15 days, then 4 drops/day for 7 days, 2 drops/day for 4 days, then 1 drop/day for 3 days to replicate the typical pattern of ocular steroid tapering used in clinic. Identical bottles were used for both licaminlimab and dexamethasone ophthalmic solutions. Randomization data were kept strictly confidential, and were accessible only to authorized personnel, until unmasking of the trial.

### Dose Selection

Licaminlimab has been tested in a multiple ascending dose study in healthy volunteers. Licaminlimab was found to be safe and well tolerated for the entire duration of drug administration (12 days) including the maximum dosing frequency of 12 drops/day for 5 days. The systemic exposure to licaminlimab was low. Eight drops/day are predicted to yield close to maximum drug exposure in the AC. These predictions are made from pharmacokinetic (PK) and pharmacodynamics modeling of licaminlimab and its surrogate molecule, ESBA105, using rabbit, cynomolgus monkey, and human PK data.

In this trial, the primary readout on visit 4, day 15 after 14 days of treatment at the highest dosing frequency is based on the response profile to topical corticosteroids, where 90% of the responders have fully responded after 2 weeks of treatment. The requirement for a dose tapering is driven by the need to taper the corticosteroids in the control arm. Licaminlimab does not require tapering but the dosing frequency will be reduced from visit 4, day 15 onward to maintain the masking (4 drops/day from day 15 to day 22 then switching to vehicle from day 22 to day 29.

### Assessments

Study visits were at days 1, 4, 8, 15, 22, 26, and 29. At each visit, other than day 26, AC cell grade was determined and a full eye examination was performed, including best corrected visual acuity (BCVA) using Early Treatment Diabetic Retinopathy Study (ETDRS) or Snellen method, slit-lamp examination, dilated funduscopy, and determination of IOP. In addition, blood samples were taken for determination of blood levels of licaminlimab and anti-licaminlimab antibodies. Patients did not attend study centers at the day 26 visit.

### Study End Points

The primary efficacy end point was responder status at day 15: response was defined as a reduction from baseline in AC cell grade of ≥2 (SUN). Prespecified secondary end points were time-to-response (i.e. time to attain decrease from baseline of ≥2 AC cell grade [SUN]), and use of rescue treatment (the rescue regimen used was at the discretion of the investigator). Exploratory end points were proportions of patients with AC cell grade (SUN) of 0 at any post-randomization visit, and AC cell grade (SUN) change from baseline at post-randomization visits. PK/immunogenicity end points were total serum licaminlimab concentrations, determined using an immunoassay with lower limit of quantitation (LLOQ) of 0.25 mg/mL, and presence of anti-licaminlimab antibodies before and after treatment. Safety was assessed in terms of ocular and non-ocular adverse events (coded using MedDRA version 17.0), IOP, BCVA, and results of slit-lamp and dilated fundus examinations.

### Statistical Analyses

The Per Protocol (PP) analysis set was the primary population for the primary efficacy analysis. In the PP analysis, subjects who received rescue medication any time before or on day 15 were considered as nonresponders. No imputation of missing data was performed for the primary analysis. The full analysis set (FAS), consisting of all randomized subjects who received any study drug, had baseline efficacy assessments, and had at least one post-baseline efficacy assessment, was used for sensitivity analysis.

The primary hypothesis was that the responder rate exceeds 30% among patients with AAU treated with licaminlimab. This threshold was chosen as the minimum necessary for continued clinical development of this product candidate. The null and alternative hypotheses were H0: πlicaminlimab ≤30% and H1: πlicaminlimab >30%, where πlicaminlimab was a responder rate at day 15 for the licaminlimab group. The hypothesis was evaluated using a Bayesian framework with the prior distribution on responder rate taken to be uniform (0, 1) distribution (a noninformative prior distribution). Descriptive statistics of the posterior distribution of the responder rate along with the 95% credible interval (one-sided) were provided. Evidence of treatment effect would be considered to be established if the posterior probability that the responder rate exceeds 30% was at least 95%, that is, if the lower limit of the 95% posterior interval of the responder rate is greater than 30%. The number and the percentage of subjects who were responders at day 15 were summarized by treatment. No inferential comparison to the dexamethasone group was planned, but the results for this treatment group are presented.

Secondary and exploratory efficacy end points were summarized with descriptive statistics by treatment group. Patients who received rescue medication before or at day 15 were categorized as nonresponders with respect to time to response. The PP set was used, with the data on assigned treatment.

### Study Oversight

At each participating site, an institutional review board or independent ethics committee reviewed and approved the clinical study protocol, informed consent form, and all other appropriate study-related documents. The study was designed and performed in accordance with the International Conference on Harmonisation Harmonised Tripartite Guidelines for Good Clinical Practice and with the ethical principles of the Declaration of Helsinki. The study was registered on ClinicalTrials.gov (NCT02482129). Patients were required to understand and sign the informed consent form.

## Results

### Patient Disposition

**Figure 1.**
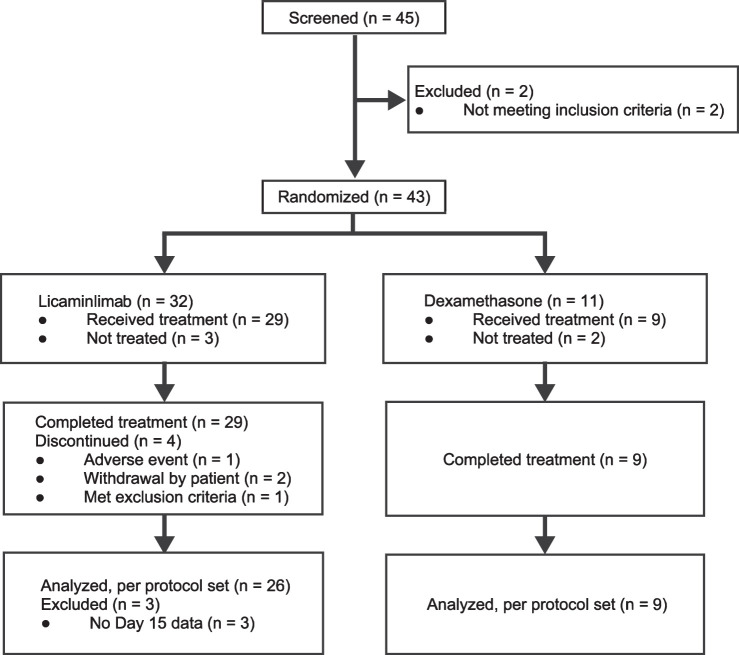
Disposition of patients (all enrolled).

Patient disposition is summarized in Figure 1. The study was conducted between July 17, 2015, and March 21, 2016. Forty-five patients were screened, and 43 were randomized. Four patients, two in each group, were not treated. All treated patients were included in the FAS and safety set. It should be noted that one patient inadvertently received dexamethasone twice before randomization to licaminlimab; this patient was analyzed as treated (i.e. included in the dexamethasone group).

### Baseline Demographics and Clinical Characteristics

Table 1 shows baseline characteristics for the per protocol set. There were some differences between groups in demographic characteristics, with higher proportions of women and black/African American patients in the licaminlimab group, and higher body weight in the dexamethasone group. The two treatment groups were generally well matched in terms of baseline clinical characteristics; mean time since diagnosis of uveitis was higher in the dexamethasone group, but this difference appeared to be due to a small number of patients with outlying values (median time since diagnosis was identical in the two treatment groups), and as no formal comparison between treatment groups was planned, this difference would not influence study outcomes.

**Table 1.** Baseline Demographic and Clinical Characteristics (Per Protocol Analysis Set)

	Licaminlimab (*N* = 25)	Dexamethasone (*N* = 10)	Overall (*N* = 35)
**Demographic characteristics**			
**Age** **,** **years**			
*N*	25	10	35
Mean (standard deviation)	53.5 (17.33)	55.6 (16.02)	54.1 (16.76)
Median (range)	52 (21-93)	50 (29-80)	50 (21-93)
**Weight** **,** **kg**			
*N*	25	9	34
Mean (standard deviation)	78.9 (20.72)	83.9 (14.25)	80.2 (19.15)
Median (range)	75 (48.1-131)	83 (63-104)	76.15 (48.1-131)
**Sex, *n* (%)**			
Male	9 (36.0)	6 (60.0)	15 (42.9)
Female	16 (64.0)	4 (40.0)	20 (57.1)
Unknown	0 (0.0)	0 (0.0)	0 (0.0)
Undifferentiated	0 (0.0)	0 (0.0)	0 (0.0)
**Race, *n* (%)**			
White	17 (68.0)	9 (90.0)	26 (74.3)
Black or African American	7 (28.0)	0 (0.0)	7 (20.0)
American Indian or Alaska Native	0 (0.0)	0 (0.0)	0 (0.0)
Asian	1 (4.0)	1 (10.0)	2 (5.7)
**Clinical characteristics**			
**AC cell grade**			
*N*	25	10	35
Mean (standard deviation)	2.4 (0.50)	2.2 (0.42)	2.3 (0.48)
Median (range)	2.0 (2-3)	2.0 (2-3)	2.0 (2-3)
**Days from diagnosis**			
*N*	25	10	35
Mean (standard deviation)	75.6 (260.34)	199.0 (626.13)	110.8 (393.47)
Median (range)	1.0 (1, 1296)	1.0 (1, 1981)	1.0 (1, 1981)
**First instance of flare, *N* (%)**	20 (80.0)	9 (90.0)	29 (82.9)

### Efficacy Outcomes

Response, defined as a two-step decrease or more from baseline in AC cell grade, was reported in 14 of 25 (56%) licaminlimab-treated patients, with one-sided 95% lower credible limit for the responder rate of 40.07%. Evidence of treatment effect was therefore considered to be established, as the lower limit of the 95% posterior interval of the responder rate was greater than 30%. In the dexamethasone group, the responder rate was 9 of 10 (90%), with one-sided 95% lower credible limit for the responder rate of 64.02% (Table 2).

**Table 2.** Responder Status at Day 15 and Descriptive Statistics for the Posterior Distribution of Responder Rate (Per Protocol Analysis Set)

	Licaminlimab (*N* = 25)	Dexamethasone (*N* = 10)
Responder, *N* (%)	14 (56.0)	9 (90.0)
Posterior distribution summaries		
Mean (standard deviation)	55.62 (9.41)	83.44 (10.18)
Posterior probability		
Responder rate >30%	1.00	1.00
Lower bound of 95% credible interval (1-sided)	40.07	64.02

Response is defined as a two-step decrease or more in AC cell grade as per Standardization of Uveitis Nomenclature (SUN).

The one-sided 95% lower credible limit for the responder rate must exceed 30%.

Patients receiving rescue treatment on or before day 15 considered nonresponders.

Time to response is shown in Table 3; 9 of 25 (36%) licaminlimab-treated patients and 7 of 10 (70%) patients in the dexamethasone group had a first response by day 4. Rescue medication was used by 6 of 25 (24%) licaminlimab-treated patients; none of the dexamethasone group used rescue medication. The proportion of patients with an AC cell grade of 0 at any post-treatment visit was similar in both treatment groups, 19 of 25 (76%) of licaminlimab patients and 8 of 10 (80%) of the dexamethasone group. The proportion of patients with AC cell grade of 0 in the licaminlimab group continued to increase up to day 29, 1 week after treatment was discontinued on day 22, and 2 weeks after the dose was halved on day 15 (Table 4). AC cell grades decreased from baseline, with median changes at day 15 of -2.0 in both treatment groups.

**Table 3.** Descriptive Statistics for First Response by Day (Per Protocol Analysis Set)

	Licaminlimab (*N* = 25)	Dexamethasone (*N* = 10)
	*N* (%)	*N* (%)
**Response day**		
Day 4	9 (36.0)	7 (70.0)
Day 8	5 (20.0)	2 (20.0)
Day 15	1 (4.0)	0 (0.0)
Nonresponder	10 (40.0)	1 (10.0)

Time of first response is the first visit when a two-step decrease or more in AC cell grade is observed. Nonresponders at each time point are patients who do not achieve at least a two-step decrease in AC cell grade at that time point. This includes, but is not limited to, patients who received rescue medication by each time point.

**Table 4.** Number (%) Patients With AC Cell Grade of Zero by Day (Per Protocol Analysis Set)

	*N* (%) Patients	
	Licaminlimab (*N* = 25)	Dexamethasone (*N* = 10)
**Day**		
Day 4	5 (20.0)	3 (30.0)
Day 8	7 (28.0)	7 (70.0)
Day 15	11 (44.0)	7 (70.0)
Day 22	14 (56.0)	6 (60.0)
Day 29	17 (68.0)	6 (60.0)
Unscheduled visit	2 (8.0)	0 (0.0)
Any post-treatment visit	19 (76.0)	8 (80.0)

Efficacy outcomes in the FAS analysis were consistent with those using the PP set.

### Safety Outcomes

Treatment-emergent adverse events (AEs) were reported in 19 of 29 (66%) of the licaminlimab group and 6 of 10 (60%) of the dexamethasone group. In both treatment groups, most AEs were related to ocular inflammation. The most common AEs in patients treated with licaminlimab were ocular events, namely AC cell, eye pain, iridocyclitis, AC flare, iris adhesions, photophobia, eye irritation, and IOP increase (Table 5). There were two patients who experienced AEs that were considered to be treatment-related by the investigators; both of these were ocular AEs. There were no non-ocular AEs that occurred in more than one patient in the licaminlimab group, and no treatment-related non-ocular AEs. The small size of the dexamethasone group precluded meaningful between-group comparisons of rates of specific AEs.

**Table 5.** Adverse Events Reported in ≥5% of Patients in Either Treatment Group (Safety Analysis Set)

	Licaminlimab (*N* = 29)			Dexamethasone (*N* = 10)		
	*N*	%	Events	*N*	%	Events
At least 1 adverse event	19	(65.5)	46	6	(60.0)	13
Adverse events in ≥5% of patients						
Anterior chamber cell	4	(13.8)	5	1	(10.0)	1
Eye pain	4	(13.8)	4	0	(0.0)	0
Iridocyclitis	3	(10.3)	3	1	(10.0)	1
Anterior chamber flare	3	(10.3)	3	0	(0.0)	0
Iris adhesions	3	(10.3)	3	0	(0.0)	0
Photophobia	3	(10.3)	3	0	(0.0)	0
Eye irritation	2	(6.9)	3	0	(0.0)	0
Intraocular pressure increased	2	(6.9)	4	0	(0.0)	0

One patient experienced a serious AE. This was a female patient, 42 years old, in the licaminlimab group, who was diagnosed with multiple sclerosis with optic neuritis on day 6. The patient discontinued participation in the study. This event was assessed by the investigator as unrelated to treatment.

**Figure 2.**
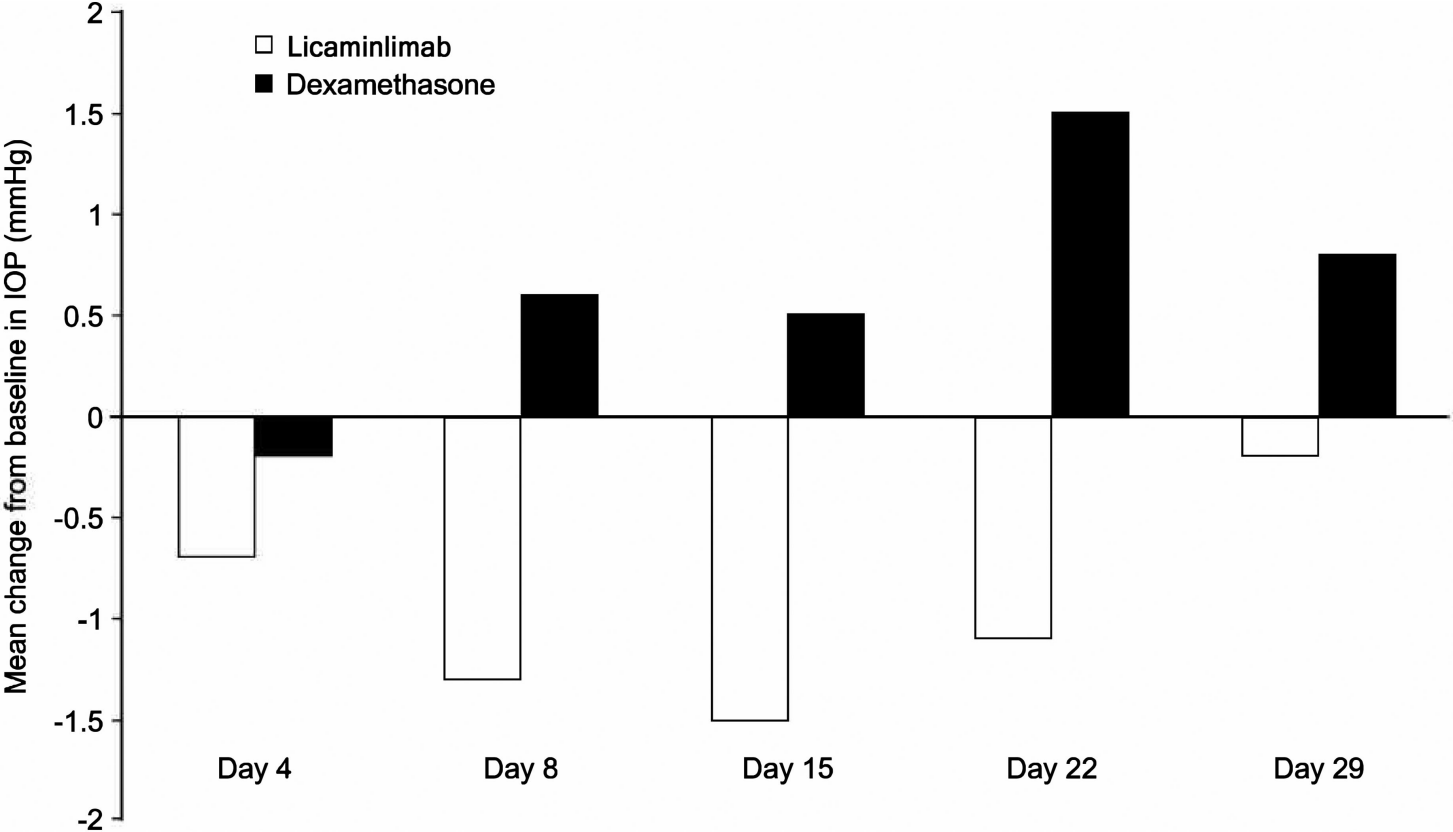
IOP change from baseline by treatment group (Safety Analysis Set).

Figure 2 shows mean change from baseline in IOP by treatment group. A mean decrease in IOP was observed in the licaminlimab group at all visits. The dexamethasone group showed mean increases from baseline of up to 1.5 mm Hg (at day 22). One licaminlimab-treated patient had an IOP increase >10 mm Hg; increases of 6 to 10 mm Hg were observed in 3 of 29 (10%) of the licaminlimab group and 2 of 10 (20%) of the dexamethasone group.

No notable changes from baseline in BCVA were observed in either treatment group. Slit-lamp examinations showed aqueous flare increases in 9 of 29 (31%) patients of the licaminlimab group and 1 of 10 (10%) patients of the dexamethasone group, increases in AC cell grade in 4 of 29 (14%) patients and 1/10 (10%) patients of each group, respectively, and keratic precipitates in 4 of 29 (14%) patients of the licaminlimab group and none of the dexamethasone group. No changes in lens parameters were observed. Few increases in fundus parameters (macula, optic nerve, retina, and vitreous assessments) were observed.

Serum concentrations of total licaminlimab were available from 29 patients at baseline, 25 at day 4, 22 at day 8, 20 at days 15 and 22, and 19 at day 29. In most patients, total licaminlimab levels were below LLOQ at all visits. At days 4, 8, 15, 22, and 29, the percentages of patients in which licaminlimab was not detectable in serum were 72.0%, 86.4%, 90.0%, 80.0%, and 100%, respectively. The highest licaminlimab serum level observed was 1.33 ng/mL (at day 22). Mean and median licaminlimab serum levels were below LLOQ at all visits other than day 22 (mean 0.25 ng/mL, median below LLOQ).

Anti-licaminlimab antibodies were detected prior to treatment in 17.2% and 30% of subjects in the licaminlimab and dexamethasone-treated dose groups, respectively. Titers in the licaminlimab group on day 1 ranged from 1.19 to 5180. The incidence of anti-drug antibodies in the licaminlimab group increased after starting treatment and with duration of dosing; the percentages of subjects in which anti-licaminlimab antibodies were detected being 19.2%, 26.1%, 55.0%, 85.0%, and 90.0% on days 4, 8, 15, 22, and 29, respectively. Corresponding titers ranged from 2.88 to 250, 1.55 to 202, 2.67 to 168, 1.38 to 13,500, and 1.24 to 7390, respectively. Anti-licaminlimab antibodies were not assessed at post-baseline visits in the dexamethasone group.

## Discussion

This pilot trial met its primary objective according to prospectively specified criteria, in the treatment of patients with AAU. Secondary and exploratory end points were supportive of the treatment effect of licaminlimab eye drops, with most patients demonstrating a reduction of ≥2 units from baseline in AC cell grade by day 8, with few patients requiring rescue therapy, with most patients achieving an AC cell grade of zero in at least one visit, and the median change from baseline in AC cell grade being equivalent to responder status.

Corticosteroid eye drops represent the current standard of care in AAU, and therefore were the therapy used in the control arm. However, no inferential comparisons between groups were planned and the 3:1 randomization ratio and small sample size make comparisons difficult. One patient received treatment with dexamethasone prior to being formally randomized. The formal randomization was to licaminlimab. However, as this patient had received dexamethasone, they continued treatment with dexamethasone. Masking was not broken for the patient, study site staff, or masked sponsor personnel, and this patient was included in the dexamethasone group for analysis. We considered this approach, rather than exclusion of the patient, to be acceptable as no formal between-group comparisons were planned, and the active control group was used to provide a benchmark as to the effectiveness and safety of current standard therapies. The response rate in the dexamethasone group was higher than that in the licaminlimab group, with a shorter time to response, and a lower proportion of patients using rescue medication. However, the proportion of patients with AC cell grade of 0 at any post-treatment visit was similar in both treatment groups. This proportion continued to increase even after the drug was tapered from 8 to 4 times a day at day 15 and discontinued at day 22. As no dose-finding studies have been performed with licaminlimab to date, it is not clear whether the dose regimen used in this pilot study is optimal, and whether higher doses would be associated with higher response rates.

On the basis of this small pilot study, licaminlimab eye drops appear to be well-tolerated. The most common AEs observed in the licaminlimab group were most likely symptoms of the underlying disease, and not related to the study medication. There were few non-ocular AEs.

Active non-infectious anterior uveitis is usually treated with topical corticosteroids. In recent phase III trials, topical treatment with difluprednate or prednisolone acetate completely suppressed the inflammation in 71% and 55%, respectively, of the patients at day 21.^28^ The topical use of corticosteroids is associated with a number of adverse ocular and systemic events. They elevate IOP in susceptible individuals (steroid responders), a phenomenon known as corticosteroid-induced ocular hypertension. In the general population, 4% to 6% are likely to be “high responders,” with elevations of IOP greater than 15 mm Hg, and approximately one-third will be “moderate responders,” with increases in IOP between 6 and 15 mm Hg after daily corticosteroid treatment for 4 to 6 weeks.^8^ Corticosteroid-induced glaucoma occurs when the IOP elevation persists and results in glaucomatous visual field loss and characteristic optic nerve changes. Another side effect of topical ocular corticosteroids is the formation of posterior subcapsular cataracts (PSCs). The incidence of PSCs is related to total topical dose and duration of treatment. Over 30% of patients receiving long-term topical ocular corticosteroid treatment develop PSC.^8^ In this study, small increases in IOP were observed at all visits other than day 4 in the dexamethasone group, but in the licaminlimab group there were small mean decreases from baseline at all time points. The reason for the decrease in IOP in the licaminlimab group is unclear, but may be associated with the treatment effect, as uveitis may be associated with increased IOP.^29^ BCVA did not show notable changes from baseline in either treatment group and funduscopy did not reveal significant findings.

Assessment of licaminlimab serum levels showed that systemic exposure following topical ocular application was minimal. The anti-TNFα antibodies currently in clinical use (in some cases for the treatment of forms of uveitis, in addition to other conditions) are administered subcutaneously or intravenously, and typically achieve serum concentrations in the µg/mL range^13^^,^^18^^–^^20^ but, in this study, the maximum concentration was around 1 ng/mL. The low serum levels observed following topical ocular application of licaminlimab could allow treatment of ocular conditions without the class effect risks associated with current systemic anti-TNFα agents.

Anti-drug antibodies (ADAs) were observed pretreatment in both groups. No further determination of ADAs was undertaken in the dexamethasone group, but in the licaminlimab group the incidence of ADAs increased after starting treatment and with duration of dosing. There was no obvious impact of anti-licaminlimab antibodies, either pre-existing or treatment emergent, on the serum concentrations of licaminlimab. Similarly, there was no obvious association between the presence of anti-licaminlimab antibodies and AEs or efficacy outcomes.

Pre-existing antibodies against therapeutic antibodies have been observed for several systemically administered biologics. This is believed to be a consequence of proteolysis of endogenous immunoglobulins and not indicative of prior treatment with a therapeutic antibody. In some patient populations, pretreatment ADA may be associated with a higher risk of development of post-treatment ADA.^30^ In patients treated with systemic anti-TNFα agents, ADA development is commonly observed and may be associated with decreased therapeutic responses.^31^ However, none of five studies included in a recent review on intravitreal administration of anti-TNFα agents to treat uveitis assessed ADAs.^32^ ADAs have been reported after intravitreal administration of an scFv antibody fragment vascular endothelial growth factor (VEGF) inhibitor (BEOVU, Novartis), although ADAs were also present in a proportion of treatment-naive patients. The effects of ADAs on efficacy and safety were unknown.^33^ As the apparent development of ADAs may be affected by the sensitivity and specificity of the assays used, timing of sample collection, sample handling, and other variables, such as underlying diseases and concomitant medication, comparison of the incidence of ADAs between drugs, or drug classes may be misleading. The clinical relevance of ADAs following topical licaminlimab treatment therefore remains unknown, although, in this study, no obvious effects were apparent.

In summary, this pilot study with topical ocular licaminlimab demonstrated the prospectively specified effect (a responder rate exceeding 30% in patients with AAU treated with licaminlimab) in the treatment of AAU, with good local tolerability (including no increase in IOP) and no obvious systemic safety issues. Systemic exposure to licaminlimab was minimal after topical ocular administration. The results of the study met the criteria for further clinical development and suggest that licaminlimab could provide an effective topical treatment for acute anterior uveitis, without the well-known safety issues of topical corticosteroids, or the class-effect risks of systemic TNFα blockade, although the optimal dose regimen has yet to be determined.
